# Surgical management of a blast-induced ankle injury complicated by myiasis using cross-leg flap reconstruction: A case report

**DOI:** 10.1016/j.ijscr.2025.111795

**Published:** 2025-08-12

**Authors:** Emal Wardak, Hamid Jamal, Hares Aref, Ajmal Wardak, Torgot Ghani

**Affiliations:** a Prof. Mussa Wardak Hospital, Kabul, Afghanistan; b Wazir Mohammad Akbar Khan (WMAK) hospital, Kabul, Afghanistan

**Keywords:** Case report, Limb salvage, Blast injury, Myiasis, Cross-leg flap, Sunlight therapy

## Abstract

**Introduction:**

Blast injuries to the lower extremities often result in extensive soft tissue damage and are prone to complications such as infection and, rarely, maggot infestation. These challenges can jeopardize limb salvage, especially in resource-limited settings. We report a successfully managed case of a neglected anterior ankle blast wound complicated by infection and myiasis, treated with a multidisciplinary approach incorporating sunlight exposure and a cross-leg flap.

**Case presentation:**

A 38-year-old woman presented with a 20-day-old anterior ankle blast injury complicated by infection, necrosis, and maggot infestation. After initially declining amputation, she was treated at our institution with serial debridement, systemic antibiotics, nutritional support, and adjunctive sunlight exposure. Due to extensive tissue loss and compromised local vascularity, a cross-leg flap supported by external fixation was selected for definitive coverage. Postoperative ischemic changes in the flap were noted but improved significantly with resumed sunlight therapy. Flap division and split-thickness skin grafting were successfully performed three weeks later, resulting in complete wound healing and functional recovery.

**Discussion:**

Maggot infestation, although rare—especially following blast trauma—demands aggressive debridement, meticulous hygiene, and strict infection control. Cross-leg flaps remain a valuable reconstructive option when local tissue transfer or free flaps are not feasible. In this case, sunlight therapy, an unconventional adjunct, showed clinical benefit by enhancing local wound care and flap viability.

**Conclusion:**

This case highlights the importance of multidisciplinary, adaptable approaches for complex limb injuries. Cross-leg flaps and non-traditional interventions like sunlight exposure may be effective strategies in low-resource settings.

## Highlights

• A rare ankle blast injury with infection and maggot infestation is reported.
• The condition is treated with debridement, cross-leg flap, and external fixation successfully.
• Post-blast maggot infestation is uncommon and poses surgical challenges.
• Cross-leg flap is a reliable limb salvage option in certain conditions.
• Sunlight aided wound care by drying tissue, reducing maggots, and odor control.

## 1 Introduction

Blast injuries are complex high-energy traumas that often result in deep, penetrating wounds with extensive soft tissue destruction, foreign body implantation, and contamination by multidrug-resistant environmental pathogens. These wounds are particularly challenging to manage in low-resource settings where limited access to advanced diagnostic and treatment options increases the risk of complications, including infection [1].

One rare but significant complication in such wounds is wound myiasis, the infestation of human tissue by Dipteran larvae (maggots) [2]. This condition typically arises in tropical or subtropical regions and is associated with poor hygiene, socioeconomic challenges, and proximity to domestic animals. Neglected wounds with foul-smelling discharge, such as diabetic ulcers or traumatic injuries, are especially susceptible. If not treated promptly, myiasis can lead to rapid tissue destruction, secondary infection, and even amputation [3].

In severe limb trauma where free flap options are contraindicated, cross-leg flaps offer a time-tested and reliable reconstructive solution, with reported survival rates approaching 100 % and pedicle division usually achieved within three weeks [4]. Adjunctively, solar spectrum ultraviolet light therapy has emerged as a potential treatment modality for infected wounds, showing promise in eliminating resistant pathogens and promoting wound healing through immune modulation [5]. Herein, we present a rare case of lower limb blast injury complicated by necrosis, polymicrobial infection, and myiasis, successfully managed with serial debridement, systemic antibiotics, sunlight therapy, and coverage via a cross-leg fasciocutaneous flap which is the first reported case in the literature. It is reported following the SCARE criteria [6].

## 2 Case presentation

An otherwise healthy, nonsmoking 38-year-old mother of five presented with a foul-smelling, purulent wound on the anterior aspect of her right ankle. She had sustained a bomb blast injury 20 days earlier, initially managed with herbal remedies in her village. The wound progressively worsened, becoming necrotic and infested with maggots. Once the underlying bone became visible, she was referred to a provincial hospital where a below-knee amputation was advised, which she declined.

**Fig. 1:**
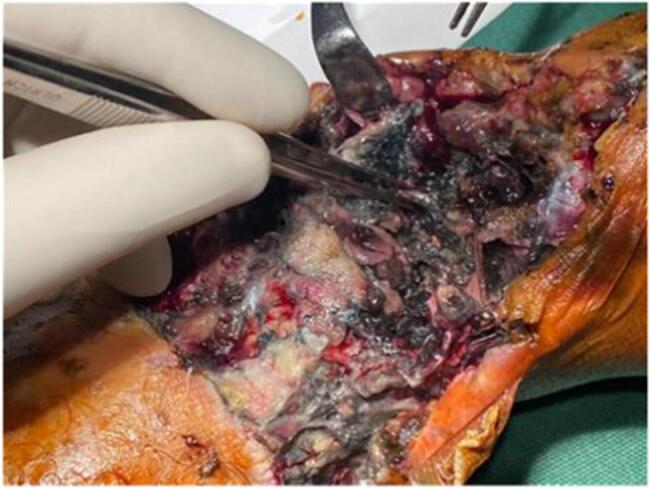
The neglected bomb blast wound which was filled with necrotic debris and infested maggots at presentation.

On examination, a 5 × 12 cm irregular wound was noted over the anterior ankle, filled with necrotic tissue and active maggot infestation, with minimal exposure of the distal tibia and talus (Fig. 1). Her vital signs were stable. Initial labs revealed hemoglobin of 8.2 g/dl, leukocyte count of 12,000/μl (45 % lymphocytes, 2 % basophils), serum albumin of 4 g/dl, and CRP of 28 mg/l. She received two units of packed red blood cells, followed by surgical debridement within 24 h. The ankle joint was immobilized with a posterior back slab, and Piperacillin-Tazobactom 4.5 g TDS with Linezolid 600 mg BID were initiated. Given the high risk of polymicrobial contamination in blast injuries and the limited value of surface cultures, routine swab cultures were not performed.

Daily care included wound irrigation, dressing changes, nutritional support, medications, and manual maggot removal. Due to poor initial response, 30-min daily sunlight exposure at 10:00 AM was started on day three. At Kabul's altitude (1797 m), with ambient temperatures of 27–35 °C and 18 % higher UV intensity, notable reductions in foul odor, discharge, and inflammation were observed. Over the next week, maggots were eradicated, necrotic tissue eliminated, and the wound bed improved. CRP declined to 4 mg/l, and the patient stabilized clinically, making her eligible for definitive surgical closure.

**Fig. 2:**
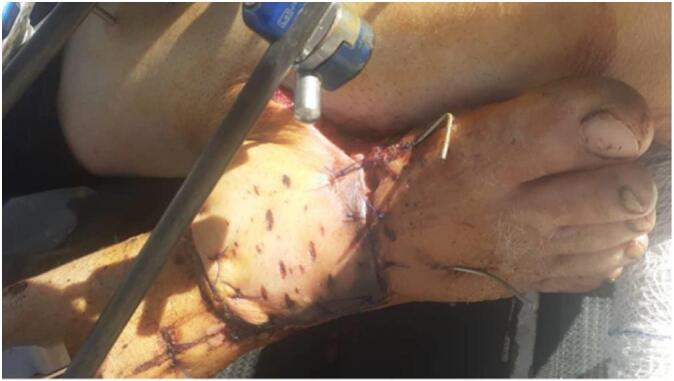
The wound site after cross-leg fasciocutaneous flap and external fixation.

Given the defect's size, location, and lack of local tissue options, a posterior tibial artery perforator-based cross-leg fasciocutaneous flap from the contralateral limb was planned. A hand-held Doppler identified a suitable perforator, confirmed intraoperatively via exploratory incision. The flap dimensions were carefully tailored to the wound size and base was narrowed to prevent pedicle kinking and enhance rotation. It was then harvested and inset tension-free with a short bridging segment. Both limbs were immobilized using an AO external fixator (Fig. 2). The donor site was grafted with a full-thickness skin graft from the ipsilateral anterior thigh. The procedure was performed in a tertiary hospital by a reconstructive orthopedic surgeon with over 20 years of experience.

**Fig. 3:**
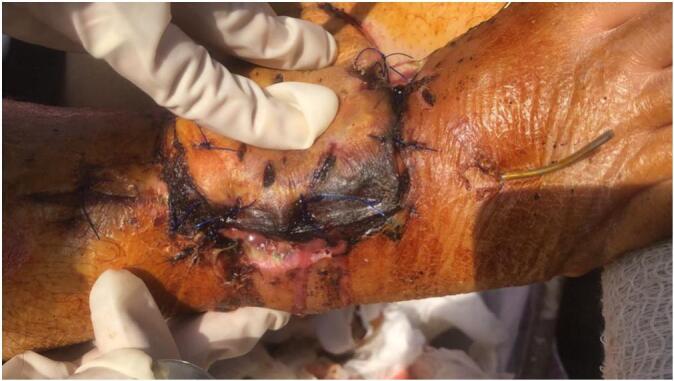
Threatened flap exhibiting poor attachment, purulent discharge and marginal necrotic changes.

In the early postoperative period, limited mobility prevented continued sunlight exposure. By postoperative day 3, marginal flap ischemia (∼1 cm) and swelling were observed (Fig. 3). After reinitiating sunlight therapy, improvement in flap color and edema was noted.

**Fig. 4:**
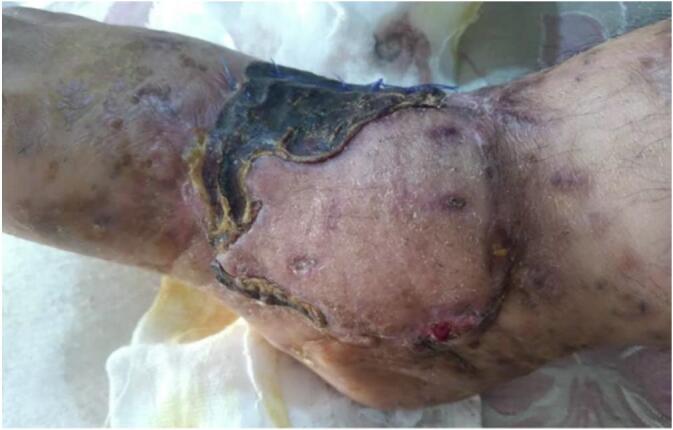
The condition of flap after its complete separation and removal of the external fixator.

By postoperative day 14, flap division was initiated and completed gradually by day 21. Necrotic edges were clearly demarcated and easily excised. The external fixator was removed (Fig. 4). A long-leg plaster back slab with a monitoring window was applied to protect the graft.

**Fig. 5:**
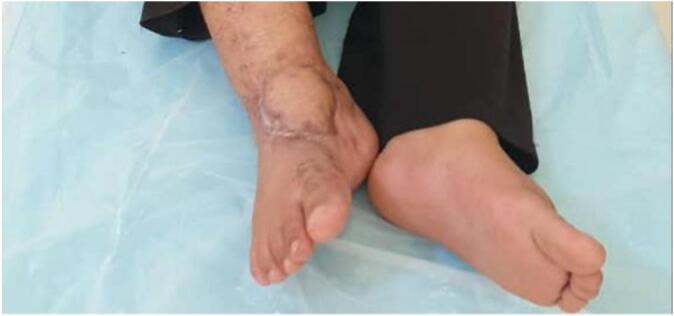
Wound condition at 3 months post-op shows complete recovery.

**Fig. 6:**
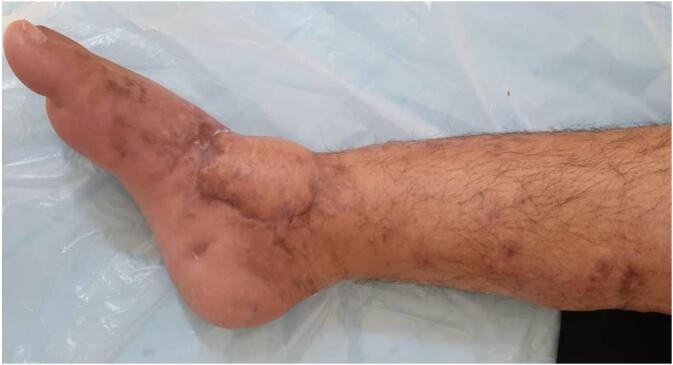
Wound condition at 3 months post-op shows complete recovery.

**Fig. 7:**
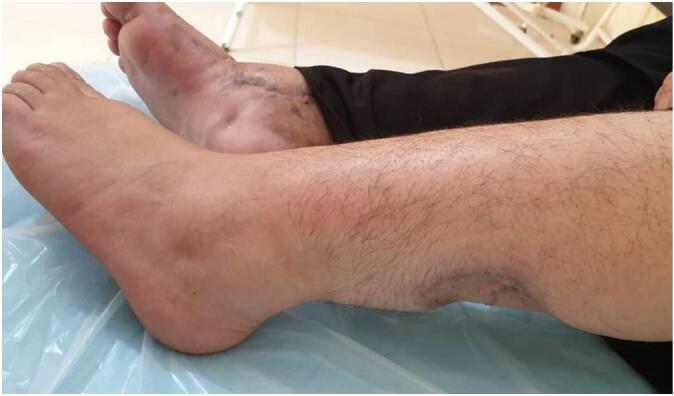
Donor site has had complete healing.

On postoperative day 31, the slab was removed, and physiotherapy and range-of-motion exercises began. The wound had healed sufficiently, and the patient ambulated without discomfort. At a three-month follow-up, she shared photographs demonstrating complete healing of both donor and recipient sites. Clinical examination confirmed full functional recovery and resolution of symptoms (Fig. 5, Fig. 6, Fig. 7). Clinical timeline of wound management is concluded in (Table 1).

**Table 1:** 

Day	Key event
Day 1	Patient presented with a neglected, maggot-infested blast wound. Decided for limb reconstruction. Labs showed anemia and elevated CRP. Received blood transfusion, underwent urgent surgical debridement, started IV Piperacillin–Tazobactam and Linezolid, and wound was immobilized.
Day 1–8	Daily wound care included maggot removal and sunlight therapy (30 min/daily), the wound condition improved, CRP declined to 4 mg/l, the wound clinically eligible for definitive surgical closure.
Day 9	Cross-leg fasciocutaneous flap surgery performed, AO external fixators applied, and skin graft applied.
Post-op day 3	Marginal ischemia and swelling observed, sunlight exposure therapy resumed.
Post-op day 4–14	Standard daily wound care continued, necrotic edges demarcated, swelling subsided, flap was ready for division.
Post-op day 14–21	Gradual flap division initiated on day 14, demarcated necrotic edges excised, on day 21 flap division gradually completed, and long leg slap applied with monitoring window.
Post-op day 31	Slab removed, physiotherapy initiated
3-month follow-up	Full recovery documented

## 3 Discussion

Blast injuries, particularly from high-velocity trauma, present considerable reconstructive challenges due to extensive soft tissue destruction and inevitable contamination. These wounds are generally considered infected at presentation, with deeply embedded debris and polymicrobial colonization by multidrug-resistant organisms. Surface cultures and gram stains often fail to reflect the true burden of infection, and decisions regarding debridement or closure are usually based on clinical judgment rather than objective criteria [1].

This patient presented with a chronic blast wound on the anterior ankle with minimal bone exposure and myiasis—a rare but serious complication in humans. While aggressive debridement, antibiotics, and flap reconstruction may salvage the limb, amputation remains inevitable in advanced cases [3]. Myiasis typically occurs when flies deposit eggs in necrotic or infected tissue, allowing larvae to feed and burrow, causing further tissue damage [2]. This condition is more prevalent in rural or tropical settings with poor hygiene and limited healthcare access. Prompt diagnosis—typically by direct visualization of larvae—followed by mechanical removal, serial debridement, irrigation and systemic antibiotics, is crucial. While the referring center recommended amputation—a risk heightened by delayed diagnosis and prolonged ineffective antibiotic treatments, which can exacerbate tissue destruction [3]—we instead pursued aggressive wound care with staged debridement and a final fasciocutaneous flap.

In cases of extensive soft tissue loss with compromised vascular supply, cross-leg flaps remain a reliable reconstructive option, particularly when free tissue transfer is not feasible. They are especially indicated in single-vessel limb perfusion, severely injured extremities with inadequate recipient veins, and polytrauma patients unsuitable for prolonged microsurgery [9].

Free flap reconstruction was not possible in this case due to delayed referral and lack of suitable recipient vessels [9]. Additionally, the risk of amputation secondary to free flap procedure's failure which can reach 57 % in such cases had to be avoided [13]. The cross-leg fasciocutaneous flap was chosen due to its high success rate (up to 94 %), shorter operative time, minimal donor-site morbidity, fewer aesthetic concerns, and low need for secondary revisions [14]. To improve flap viability, a delay procedure is routinely employed. While techniques delay may vary, our preferred approach involves initiating flap delay around the second postoperative week, followed by staged division, typically completed by gradually by the end of third week [9].

We observed the positive effect of sunlight on infection control in this particular case. Controlled solar spectrum exposure has been shown to reduce microbial burden—including drug-resistant pathogens—and enhance healing by modulating antioxidant and immune responses [[10], [11], [12]]. While case reports exist on myiasis on neglected wounds, cross leg flap salvage in trauma, and sunlight therapy in wound healing, none have documented concurrent use of all modalities in a blast-injured maggot-infested lower extremity.

## 4 Conclusion

This case underscores the multifaceted challenges of managing complex blast injuries in resource-limited settings, particularly when compounded by rare complications. Through a strategic combination of serial surgical debridement, systemic antimicrobial therapy, adjunctive sunlight therapy, and definitive coverage with a cross-leg fasciocutaneous flap, limb salvage was successfully achieved. To our knowledge, this is the first reported case to utilize all these modalities in concert, offering valuable insight into adaptable and pragmatic approaches for limb reconstruction in complicated conditions. Further research is warranted to establish standardized protocols for integrating sunlight therapy and cross-leg flap techniques in similarly complex wounds.

## Consent

Written informed consent was obtained from the patient for publication of this case report and accompanying images. A copy of the written consent is available for review by the Editor-in-Chief of this journal on request.

## Ethical approval

Ethical approval was not required for this case report in accordance with the policies of Prof. Mussa Wardak Hospital. Written informed consent was obtained from the patient for both the treatment and the publication of this report, including the use of clinical images, due to the rare nature of the intervention.

## Guarantor

Dr. Emal Wardak and Dr. Hamid Jamal are the guarantors of this article and take responsibility for the integrity of the whole work as a whole.

## Funding

The authors received no funding for the writing of this article.

## Author contribution

**Emal Wardak**: Conceptualization, Validation, Investigation, Supervision

**Hamid Jamal**: Data curation, Writing-original draft, Visualization

**Hares Aref**: Resources, Data curation, Supervision

**Ajmal Wardak**: Data curation, Writing-Review and editing

**Torgot Ghani**: Writing-review and editing

All authors have seen and approved the final manuscript.

## Conflict of interest statement

All authors declare no conflict of interest.
